# Cocrystallization of ubiquitin–deubiquitinase complexes through disulfide linkage

**DOI:** 10.1107/S2059798323008501

**Published:** 2023-10-25

**Authors:** Kristos I. Negron Teron, Chittaranjan Das

**Affiliations:** a Department of Chemistry, Purdue University, 560 Oval Drive, West Lafayette, IN 47907, USA; UNSW Sydney, Australia

**Keywords:** deubiquitinases, ubiquitin, deubiquitinase–ubiquitin complexes, disulfide-linked protein complexes

## Abstract

Structural characterization of the recognition of ubiquitin (Ub) by deubiquitin­ases (DUBs) has largely relied on covalent complexation of the DUB through its catalytic cysteine with a Ub C-terminal electrophile. The Ub electrophiles are accessed through intein chemistry in conjunction with chemical synthesis. Here, it was asked whether DUB–Ub covalent complexes could instead be accessed by simpler disulfide chemistry using a Ub cysteine mutant in which the last glycine has been replaced with a cysteine. The Ub cysteine mutant displayed a wide variability in disulfide formation across a panel of eukaryotic and prokaryotic DUBs, with some showing no detectable reaction while others robustly produced a disulfide complex. Using this approach, two disulfide-linked ubiquitin-bound complexes were crystallized, one involving the *Legionella pneumophila* effector SdeA DUB and the other involving the *Orientia* effector OtDUB. These DUBs had previously been crystallized in Ub-bound forms using the C-terminal electrophile strategy and noncovalent complexation, respectively. While the disulfide-linked SdeA DUB–Ub complex crystallized as expected, in the OtDUB complex the disulfide bond to the Ub mutant involved a cysteine that differed from the catalytic cysteine. Disulfide formation with the SdeA DUB catalytic cysteine was accompanied by local distortion of the helix carrying the active-site cysteine, whereas OtDUB reacted with the Ub mutant using a surface-exposed cysteine.

## Abstract 2

The crystal structures of two disulfide-linked ubiquitin-bound bacterial deubiquitinases reveal the mode of ubiquitin binding. These structures show that disulfide linking is an effective strategy for capturing covalent ubiquitin–deubiquitinase complex structures.

## 1. Introduction

Protein ubiquitination involves the post-translational covalent attachment of the 76-residue eukaryotic protein ubiquitin (Ub) to, most commonly, lysine residues of target proteins via an isopeptide bond that links the last glycine of Ub to the ɛ-amino group of the target lysine. Attachment of a single Ub, or monoubiquitination, can serve as a regulatory signal, but protein targets are usually polyubiquitinated through successive extension of the Ub–Ub linkage using any of the seven lysines (or the Met1 α-amino group) of the modifier itself. In its simplest form, polyubiquitination can arise with eight topologically distinct linkage types in homotypic chains, which are chains produced by repeated use of the same Ub amino group. Additionally, the use of different lysines during chain extension, along with branching, can give rise to ubiquitination patterns that represent complex signals constituting what has been referred to as the ubiquitin code. The writers of the code are the E1, E2 and E3 enzymes, which function in coordination to first activate Ub by an ATP-requiring reaction catalyzed by the E1 enzyme, followed by transfer of the activated Ub to the catalytic cysteine of a ubiquitin-conjugating E2 enzyme and finally to a substrate lysine through the intermediacy of a Ub E3 ligase (Komander & Rape, 2012 ▸). The readers are one of several ubiquitin-binding domains found in receptor proteins that recognize the Ub tag and shepherd the modified protein to its biological fate (Husnjak & Dikic, 2012 ▸). Last but not least are erasers, or deubiquitin­ases (DUBs), that remove Ub from ubiquitinated proteins or trim and disassemble polyubiquitin chains, regulating the signal (Komander & Rape, 2012 ▸; Eletr & Wilkinson, 2014 ▸; Nijman *et al.*, 2005 ▸).

The 90 or so eukaryotic DUBs can be classified into seven DUB families belonging to two broad mechanistic classes: cysteine proteases and metalloproteases (Komander & Rape, 2012 ▸; Eletr & Wilkinson, 2014 ▸; Nijman *et al.*, 2005 ▸). Within the cysteine protease class, the eukaryotic DUBs are divided into six groups based on the structure of their catalytic domain: the ubiquitin carboxy-terminal hydrolase (UCH) family, the ubiquitin-specific protease (USP) family, the ovarian tumor (OTU) family, the Machado–Joseph domain (MJD) family, the motif interacting with Ub-containing novel DUB (MINDY) family and the zinc finger with UFM1-specific peptidase domain protein (zUFSP) family groups (Komander & Rape, 2012 ▸; Hewings *et al.*, 2018 ▸; Kwasna *et al.*, 2018 ▸), whereas the JAMM/MPN (JAMM) family of metalloenzymes are the only group of DUBs that use a zinc-dependent, thermolysin-like mechanism in their hydrolysis reaction (Sato *et al.*, 2008 ▸; Shrestha *et al.*, 2014 ▸).

Mechanistic understanding of DUBs requires their co-crystal structure with Ub to delineate the intermolecular recognition. A widely used strategy for capturing the Ub-bound DUB complex relies on a Ub-derived irreversible inhibitor that carries a C-terminal electrophile warhead exactly in place of the scissile peptide bond immediately following Gly76, the site of DUB cleavage. The Ub moiety, captured through covalent tethering with the catalytic cysteine, invariably occupies the so-called distal site (or S1 site; Zhang & Das, 2023 ▸; Borodovsky *et al.*, 2002 ▸). Ub at the proximal site (S1′ site) has been captured using diubiquitin probes with a suitably placed internal electrophile between the Ub units (Li *et al.*, 2014 ▸; Haj-Yahya *et al.*, 2014 ▸). However, the synthesis of such probes requires special expertise. We reasoned that disulfide linking may provide an easy-to-access alternative that does not involve any specialized synthetic methods. For example, placement of cysteine in place of Ly63 of Ub should permit the capture of Ub in the S1′ site of Lys63-specific cysteine DUBs. Towards this goal, we sought to determine whether cysteine substitution and subsequent disulfide formation is feasible at all, which we wanted to test with the Gly76-to-Cys Ub mutant, which is meant to react with the catalytic cysteine and allow the capture of Ub at the S1 site of the DUB.

Here, we explored disulfide-bridge formation as a strategy for producing a stable DUB–Ub complex amenable to crystallization. We envisioned that placement of a cysteine residue in Ub, which otherwise lacks this residue in the native form, in place of the last glycine may permit covalent capture of DUB–Ub complexes, allowing structural characterization. To this end, the Gly76-to-Cys mutant of Ub (Ub^G76C^) was tested for its ability to form a disulfide bond with the catalytic cysteine of a group of DUBs. Two previously crystallized complexes of bacterial DUBs from pathogenic organisms were subjected to crystallization as their Ub^G76C^ complex, allowing an assessment of the disulfide strategy. While the SdeA DUB, an effector protein from the arsenal of *Legionella pneumophila* (Sheedlo *et al.*, 2015 ▸, 2021 ▸), reacted with the Ub cysteine in the expected manner showing Ub binding at the S1 site, the effector DUB from *Orientia tsutsugamushi* (OtDUB; Berk *et al.*, 2020 ▸) preferentially reacted with a noncatalytic cysteine.

## 2. Methods

### 2.1. Protein expression and purification

OtDUB^1–259^ cloned into a pET-28a vector was obtained from Mark Hochtrasser (Yale University) and transformed into *Escherichia coli* strain BL21(DE3) (Novagen). The *E. coli* BL21(DE3) cells were grown in lysogeny broth (LB) medium at 37°C to an OD_600_ of 0.6, cooled to 18°C and induced overnight with the addition of 0.35 m*M* isopropyl β-d-1-thiogalactopyranoside (IPTG). Cells from a 6 l culture were resuspended in phosphate-buffered saline (PBS) supplemented with 400 m*M* KCl (binding buffer). The resuspended cells were lysed through a combination of lysozyme and high pressure using a French press. The lysate was centrifuged for 1 h at 100 000*g* at 4°C and the supernatant was passed through a self-packed column of 5 ml Ni–NTA resin (Qiagen) pre-equilibrated with binding buffer. The resin was then washed with five column volumes of binding buffer to wash off unbound protein. The column was further washed in two steps with binding buffer supplemented with 10 and 25 m*M* imidazole. The protein was eluted from the column using elution buffer (1× PBS supplemented with 400 m*M* KCl and 300 m*M* imidazole). The eluted protein was dialyzed in two changes of binding buffer at 4°C to remove excess imidazole. The dialyzed protein was then concentrated to 21 mg ml^−1^ and buffer-exchanged into a buffer consisting of 50 m*M* Tris pH 7.4, 100 m*M* NaCl (cross-linking buffer). The purity of the enzyme was verified by sodium dodecyl sulfate polyacrylamide gel electrophoresis (SDS–PAGE).

Ubiquitin with a C-terminal glycine-to-cysteine mutation (Ub^G76C^) was cloned into a pRSET-A vector and transformed into *E. coli* strain BL21(DE3). The *E. coli* BL21(DE3) cells were grown in LB medium at 37°C to an OD_600_ of 0.6, cooled to 18°C and induced overnight with the addition of 0.35 m*M* IPTG. Cells from a 3 l culture were resuspended in 50 m*M* sodium acetate pH 4.5 (buffer *A*). The resuspended cells were initially lysed with lysozyme and by heating at 80°C for 30 min. The lysate was centrifuged for 1 h at 100 000*g* at 4°C and the supernatant was passed through a self-packed column of SP Sepharose Fast Flow resin (GE Healthcare) that had been pre-equilibrated with five column volumes of buffer *A*. After collecting the flowthrough, the column was washed with an additional five column volumes of buffer *A* to remove any unbound protein. To elute the bound protein, buffer *A* supplemented with increasing amounts of NaCl ranging from 100 m*M* to 1 *M* was used as a gradient to separate it from other potential contaminants. The fractions containing pure Ub^G76C^, confirmed by SDS–PAGE, were pooled, concentrated and buffer-exchanged into 0.1 *M* sodium phosphate buffer pH 8 (DTNB reaction buffer).

For SdeA DUB (Puvar *et al.*, 2019 ▸), UchL1, UchL3, Zup-1, MINDY-1, LotA, LotB, ElaD and ChlaDUB2, *E. coli* BL21(DE3) cells were grown in LB medium at 37°C to an OD_600_ of 0.6–0.8, cooled to 18°C and induced overnight with the addition of 0.35 m*M* IPTG. The cells from a 6 l culture were resuspended in binding buffer. The resuspended cells were then lysed using a combination of lysozyme and and high pressure using a French press. The lysate was centrifuged for 1 h at 70 000*g* at 4°C and the supernatant was passed through a self-packed column of 5 ml Glutathione Sepharose resin (GE Healthcare) pre-equilibrated with binding buffer. The resin was then washed with 20 column volumes of binding buffer to wash off unbound protein. The bound fusion proteins were eluted with GST elution buffer (binding buffer with the addition of 10 m*M* reduced glutathione). The protein was dialyzed against two changes (4 l each) of GST dialysis buffer at 4°C, with the addition of PreScission protease, to cleave off the GST tag. The dialyzed protein was then passed through the GST column to remove cleaved GST and PreScission protease from the protein solution. The proteolyzed protein was then further purified by size-exclusion chromatography to remove any residual GST. The purified sample was then concentrated, aliquoted and stored at −80°C. The purity of the enzymes from every stage of expression and purification were monitored by SDS–PAGE.

For OTULIN, *E. coli* BL21(DE3) cells were grown in LB medium at 37°C to an OD_600_ of 0.6–0.8, cooled to 16°C and induced overnight with the addition of 0.35 m*M* IPTG. Cells from a 6 l culture were resuspended in PBS supplemented with 400 m*M* KCl (binding buffer). The resuspended cells were lysed using a combination of lysozyme and high pressure using a French press. The lysate was centrifuged for 1 h at 100 000*g* at 4°C and the supernatant was passed through a self-packed column of 5 ml Ni–NTA resin (Qiagen) pre-equilibrated with binding buffer. The resin was then washed with five column volumes of binding buffer to wash off unbound protein. This column was further washed with binding buffer supplemented with 10 and 25 m*M* imidazole. The protein was eluted from the column using elution buffer (1× PBS supplemented with 400 m*M* KCl and 300 m*M* imidazole). The eluted protein with SENP2 protease was dialyzed in two changes of binding buffer at 4°C to remove excess imidazole. The dialyzed protein was then passed through the Ni–NTA resin to remove the His-SUMO tag. The proteolyzed protein was concentrated and buffer-exchanged into a buffer consisting of 50 m*M* Tris pH 7.4, 100 m*M* NaCl (cross-linking buffer). The purity of the enzyme was verified by SDS–PAGE.

### 2.2. Disulfide cross-link screening

Disulfide cross-linking was based on a previously described procedure (Pruneda *et al.*, 2016 ▸). A 10 m*M* stock of 5,5′-dithio-bis(2-nitrobenzoic acid) (DTNB) was made by dissolving DTNB in DTNB reaction buffer. Ubiquitin^G76C^ was mixed with resuspended DTNB at a final reaction concentration of 250 µ*M* ubiquitin and 2 m*M* DTNB overnight at 4°C to form 2-nitro-5-thiobenzoic acid (TNB)-labeled ubiquitin (Ub^76^-TNB). The Ub^76^-TNB was buffer-exchanged into cross-linking buffer to remove excess DTNB and release 2-nitro-5-thiobenzoic acid from the solution. The buffer-exchanged Ub^76^-TNB was mixed with different DUBs at an equal molar ratio in cross-linking buffer and left to react for 1 h at room temperature with shaking end over end. The resulting complex was subsquently buffer-exchanged in cross-linking buffer to remove the released TNB. The formation of cross-linking was monitored via SDS–PAGE.

### 2.3. Crystallization

Purified SdeA DUB was mixed with Ub^G76C^-TNB adduct in a 1:2 molar ratio and allowed to incubate overnight at 4°C. After this, the complex was purified by cation-exchange chromatography (Mono Q, GE Healthcare) followed by size-exclusion chromatography (Superdex 75, GE Healthcare). The purified complex was concentrated to 30 mg ml^−1^ and crystallized in hanging drops consisting of 2.8 *M* sodium acetate–HCl pH 7 after 18 days at room temperature. A complete data set was collected at 2.81 Å resolution from a single crystal on the NE-CAT beamline 24-ID-E (λ = 0.97918) at the Advanced Photon Source (APS), Argonne National Laboratory.

OtDUB^1–259^ disulfide was mixed with Ub^G76C^-TNB (in a nearly 1:1 molar ratio) and left overnight at 4°C. The reaction mixture was concentrated to 36 mg ml^−1^ and used directly in crystallization without further purification steps. Crystals were grown by the hanging-drop vapour-diffusion method at 20°C in crystallization buffer consisting of 0.16 *M* magnesium chloride, 0.08 *M* Tris pH 8.5, 24% PEG 8000, 20% glycerol after one day at room temperature. To confirm that the crystals obtained were of the disulfide complex, crystals were dissolved in both 5× reducing and 5× nonreducing SDS loading dye and analyzed using SDS–PAGE. A complete data set was collected to 1.85 Å resolution from a single crystal on the NE-CAT beamline 24-ID-C (λ = 0.97918) at the APS, Argonne National Laboratory.

### 2.4. Structure determination

Diffraction data sets were collected at the APS, Argonne National Laboratory and were processed using *HKL*-2000 (Otwinowski & Minor, 1997 ▸). The structure of SdeA DUB disulfide-linked with Ub^G76C^ was determined by maximum-likelihood molecular replacement using *Phaser* (McCoy *et al.*, 2007 ▸) within the *Phenix* suite (Liebschner *et al.*, 2019 ▸). The structure was then solved by molecular replacement using the SdeA DUB (Sheedlo *et al.*, 2015 ▸) structure (PDB entry 5crb) and ubiquitin (Vijay-kumar *et al.*, 1987 ▸; PDB entry 1ubq) as models. The asymmetric unit contained two molecules, which were SdeA DUB and Ub^G76C^. The structure was subjected to multiple steps of model building with *Coot* (Emsley *et al.*, 2010 ▸) and refinement with *Phenix*, which resulted in a final structure at 2.81 Å resolution (Table 1 ▸). The final structure was validated using *MolProbity* (Chen *et al.*, 2010 ▸) and deposited in the Protein Data Bank.

The OtDUB–Ub^G76C^ crystal data were indexed, integrated and scaled using *X-ray Detector Software* (*XDS*; Kabsch, 2010 ▸), *AIMLESS* (Evans & Murshudov, 2013 ▸) and multiple programs from the *CCP*4 suite (Agirre *et al.*, 2023 ▸), all integrated into the *RAPD* auto-processing program at NE-CAT, in the orthorhombic space group *P*22_1_2_1_. The structure was solved by molecular replacement using the native OtDUB structure (Berk *et al.*, 2020 ▸; PDB entry 6ups) and ubiquitin (Boudreaux *et al.*, 2010 ▸; PDB entry 1ubq) as models. The asymmetric unit consisted of one OtDUB molecule and one ubiquitin molecule. Sequential rounds of model building with *Coot* (Emsley *et al.*, 2010 ▸) and refinement with *Phenix* were used to arrive at the final structure, which was then validated using *MolProbity* and deposited in the Protein Data Bank.

## 3. Results

### 3.1. The reactivity of a panel of DUBs towards Ub^G76C^ shows significant variability

**Figure 1:**
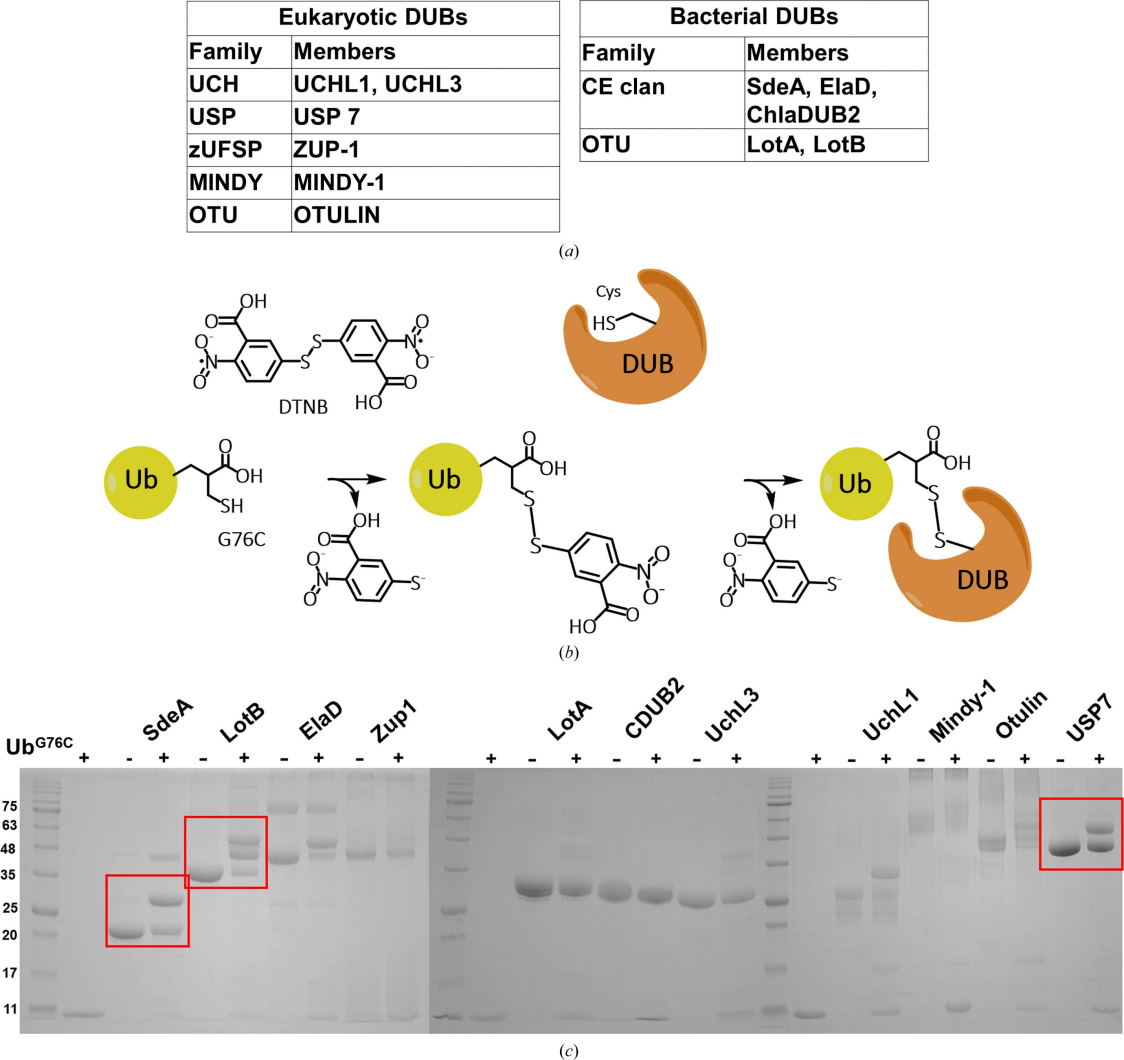
Disulfide-bridge formation as a strategy to capture disulfide-linked DUB–Ub structures. (*a*) A list of the DUBs tested for disulfide reaction. Eukaryotic and prokaryotic DUBs are grouped according to their families. (*b*) A schematic of Ub^G76C^ (yellow) disulfide-bridge formation with a target DUB (orange) utilizing DTNB. (*c*) SDS–PAGE analysis under nonreducing conditions of the reaction mixtures of DUBs with the Ub^G76C^–TNB adduct. The red boxes indicate examples of DUBs reacting with the Ub reagent.

Hydrolytic release of Ub from a target lysine involves nucleophilic attack of the catalytic cysteine of DUB at the isopeptide bond at Gly76 of Ub that links the lysine-bearing leaving group (Boudreaux *et al.*, 2012 ▸). Crystal structures of DUBs have shown that the C-terminal Gly75-Gly76 segment of Ub is held in a narrow cleft in the active-site area where the amide carbonyl of Gly76 is positioned within attacking distance of the catalytic cysteine (Boudreaux *et al.*, 2010 ▸). This conserved stereochemical feature enables DUBs to recognize and precisely cleave the target isopeptide bond after the diglycine motif. Taking advantage of this unique DUB–Ub recognition feature, we introduced a cysteine residue in place of Gly76 in Ub for engagement with the catalytic cysteine of DUB. However, the placement of this substituent may hinder binding through steric effects. To test the feasibility of cysteine placement at this location for disulfide chemistry, we used a panel of cysteine DUBs from different families, including some recently described prokaryotic examples (Pruneda *et al.*, 2016 ▸; Hermanns & Hofmann, 2019 ▸), and screened for their ability to produce a disulfide adduct with the Ub cysteine mutant (Fig. 1 ▸ *a*). For efficient disulfide formation, Ub^G76C^ was reacted with Ellman’s reagent (DTNB) to produce a disulfide-linked 2-nitro-5-thiobenzoic acid (TNB) adduct (Lorenz *et al.*, 2016 ▸) and, after washing off the excess reagent, the adduct was treated with each DUB in our list (Fig. 1 ▸ *b*). Within an hour at room temperature, some DUBs showed a robust reaction, producing a distinct band on nonreducing SDS–PAGE corresponding to a ubiquitin adduct, while others showed no discernible activity. For example, the *Legionella* OTU DUB LotB (Ma *et al.*, 2020 ▸) showed a robust reaction (showing two closely migrating adduct bands), whereas LotA (Warren *et al.*, 2023 ▸), another OTU DUB from the same organism, produced no detectable band. On the other hand, the CE-clan DUB from the same organism, SdeA DUB, reacted readily with the ubiquitin mutant, whereas another CE-clan DUB from *Chlamydia trachomatis* (CDub2; Hausman *et al.*, 2020 ▸) showed no reaction. OTULIN (Keusekotten *et al.*, 2013 ▸) and MINDY (Kwasna *et al.*, 2018 ▸), two eukaryotic DUBs, produced smearing in the nonreducing gel, preventing a clear analysis of their disulfide product (Fig. 1 ▸ *c*). Overall, these results indicate that disulfide formation with Ub^G76C^ can vary widely across different DUBs.

### 3.2. Structure of the SdeA DUB domain in complex with ubiquitin captured by disulfide chemistry

**Figure 2:**
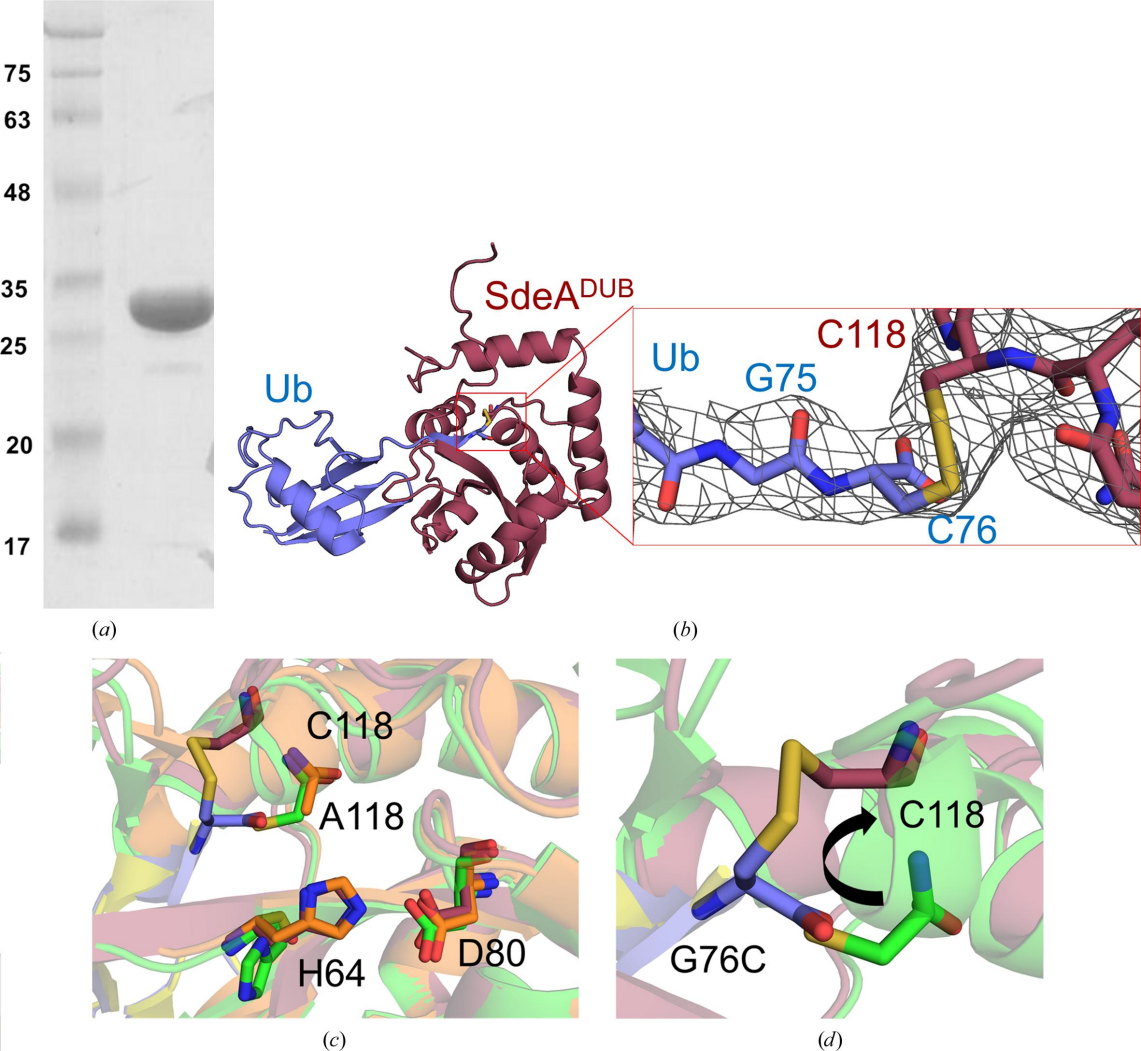
Structure of the disulfide-linked SdeA DUB–Ub^G76C^ complex. (*a*) SDS–PAGE (nonreducing) analysis of a purified sample of SdeA DUB in a disulfide-linked complex with Ub^G76C^. (*a*) Ribbon representation of the crystal structure of the disulfide-linked complex of Ub^G76C^ (slate) and SdeA (raspberry). Right: enlargement of the boxed region shows that there is continuous electron density (2*F*_o_ − *F*_c_ at 1σ) between the C-terminal cysteine of Ub^G76C^ and the catalytic cysteine of SdeA DUB. (*c*) Catalytic triad of SdeA DUB compared between the disulfide-linked structure, the Ub-VME-bound structure (lime green) and the apo structure (PDB entry 5crb, C118A mutant; orange). (*d*) Superposition of the Ub-VME-bound structure (green) and the disulfide-linked SdeA DUB–Ub complex structure (raspberry). The G76C residue is highlighted in stick representation in slate. The movement of the catalytic cysteine and the unfurling of a helical turn (that carries the catalytic cysteine) to accommodate the disulfide bridge between Cys76 of Ub and Cys118 of SdeA is shown by an arrow. The cysteine (green sticks) in the SdeA DUB–Ub-VME complex represents its native-like conformation.

Since SdeA DUB showed robust disulfide formation, we sought to crystallize the disulfide complex to characterize the binding and compare it with previously solved crystal structures of the same DUB with the Ub electrophile inhibitor ubiquitin vinyl methyl ester (Ub-VME; PDB entry 5cra; Sheedlo *et al.*, 2015 ▸). The disulfide adduct was purified by ion-exchange chromatography (using buffers without a reducing agent) and subjected to crystallization trials (Fig. 2 ▸ *a*). Crystals obtained from a condition lacking any reducing agent diffracted to 2.83 Å resolution and belonged to space group *P*3_2_21 (see Section 2; PDB entry 8efw). The structure was solved using molecular replacement (MR) with SdeA DUB and wild-type Ub as search models, assuming that the crystals belonged to the two-protein complex. The MR search yielded a solution that contained the expected 1:1 complex of the DUB and Ub. A difference electron-density (*F*_o_ − *F*_c_) map calculated after the MR search showed residual electron density extending from the DUB catalytic cysteine, which we interpreted to correspond to the disulfide bond linking the cysteine residue of Ub^G76C^. Further refinement after rounds of rebuilding and model adjustment allowed the placement of a disulfide bond into this density (Fig. 2 ▸ *b*).

**Figure 3:**
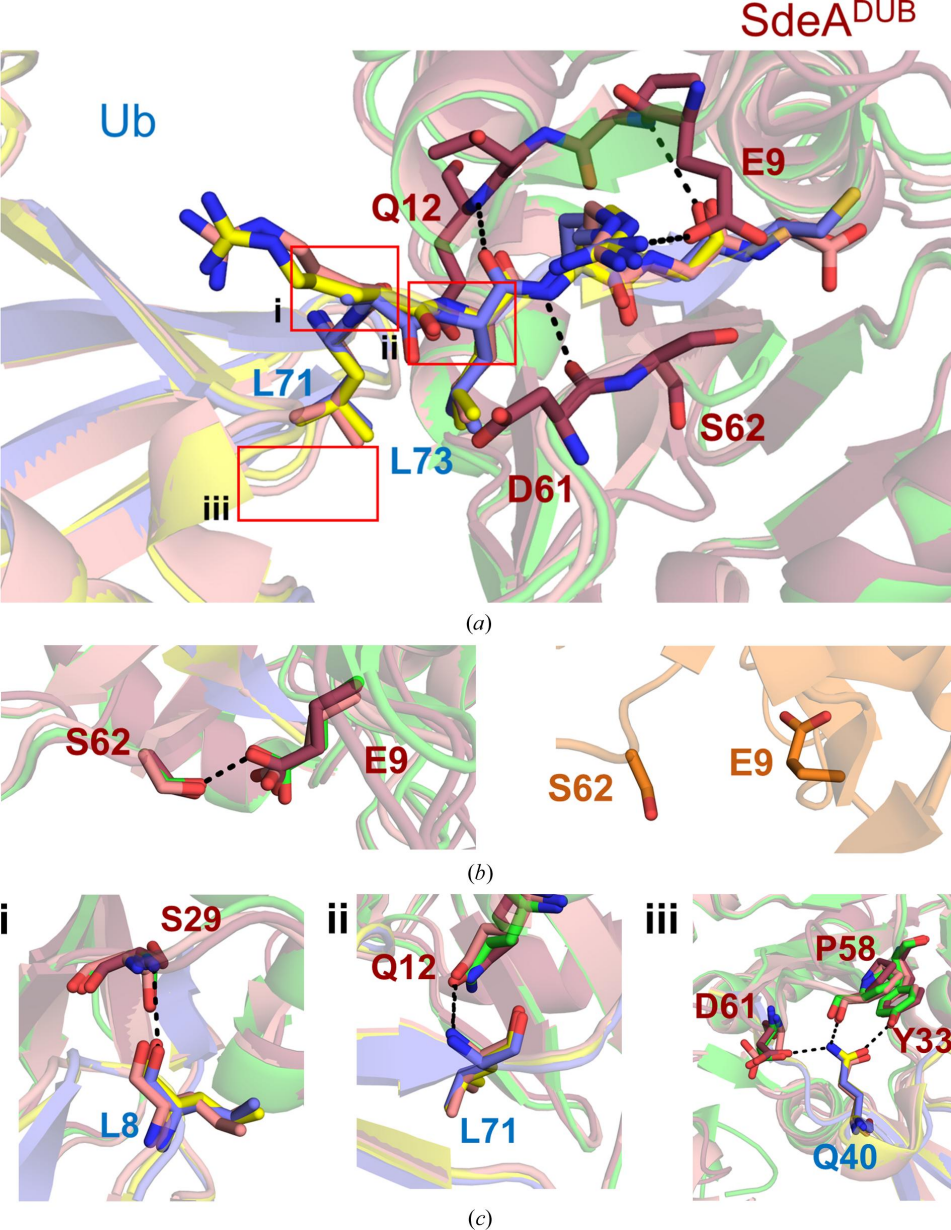
Interactions with Ub are preserved in the disulfide-linked structure. (*a*) Superposition of the SdeA DUB complex with Ub in the disulfide-linked form (raspberry and slate), the Ub-VME-bound form (green and yellow) and the Ub product-bound form (pink and orange). The C-terminal tail of Ub (from Leu71 to Gly75) is shown as sticks. Active-site SdeA DUB residues that form β-sheet-like interactions with the tail are shown as raspberry sticks. The hydrogen-bond network formed between the C-terminal tail of Ub and SdeA DUB is indicated as dashed lines. The red boxes indicate the regions depicted in (*c*). The numbering of the boxes corresponds to the numbering in (*c*). (*b*) Left: the hydrogen bond (dashed line) between SdeA DUB residues Glu9 and Ser62 is conserved in the VME-bound, product-bound and disulfide-bound structures. Right: in the apo form of SdeA DUB (orange) this interaction is not observed, indicating an open active site. (*c*) Hydrogen-bonding interactions (dashed lines) in other areas distal to the active site that were observed between Ub and SdeA DUB in the Ub-VME-bound and product-bound structures are maintained in the disulfide-linked structure.

Apart from local distortion of the SdeA DUB near the catalytic cysteine (discussed below), the structure of the complex showed striking similarity to the Ub-VME-bound structure and the Ub product-bound structure (Fig. 2 ▸ *b* and Supplementary Fig. S1). The C-terminal tail of Ub^G76C^ is held in the active-site cleft through several backbone hydrogen bonds, most of which remain the same compared with the other two structures (Fig. 3 ▸ *a* and Supplementary Fig. S4*a*). The free carboxylate at Cys76 points towards the catalytic histidine, much like the same group of Ub in the product-bound complex (Supplementary Fig. S2*a*). The tail, while in a β-sheet arrangement with two strands from either side of the cleft, is embraced from the top by a Glu–Ser hydrogen bond which has previously been noted to behave as a flap that opens and closes during substrate binding and product release (Fig. 3 ▸ *b*). This observation indicates that the cysteine mutation at Gly76 does not prevent the entry of the C-terminal Ub tail into the active-site area when the flap opens. Distal from the active site, the interactions with Ub remained largely the same compared with the other two structures. For example, Gln40 of ubiquitin makes a hydrogen-bond network with Tyr33 and Asp61 of SdeA, and Leu8 forms hydrogen bonds to the backbone and side chain of Ser29 (Fig. 3 ▸ *c* and Supplementary Fig. S4*a*).

### 3.3. Disulfide bonding to catalytic cysteine is accompanied by local distortion to accommodate the disulfide bridge

The C^α^ atom of Cys76 of the Ub^G76C^ mutant closely overlaps with the same atom of Gly76 in the Ub product-bound structure, with the free carboxylates pointing down in the same direction towards the catalytic histidine, as alluded to previously (Supplementary Fig. S2*a*). The thiol group of Ub^G76C^ points up towards the empty space in the active-site cavity, which appears to pull the DUB catalytic cysteine away from its preferred position as observed in the Ub-VME-bound structure (Fig. 2 ▸ *d*). The alanine side chain of the SdeA DUB Cys118Ala mutant (the Ub product-bound complex was crystallized with the SdeA DUB catalytic cysteine mutated to alanine) also points in the same direction as the cysteine in the Ub-VME-bound structure. The movement of the catalytic cysteine by ∼4.6 Å (C^β^–C^β^ distance) to engage with the cysteine of Ub in a disulfide bond induces a local conformational rearrangement, which seems to be necessary, partly due to having a substituent on the C^α^ atom of residue 76 and partly due to the aforementioned relocation of the DUB catalytic cysteine (Fig. 2 ▸ *d* and Supplementary Fig. S2*b*). The resulting disulfide adopts a somewhat standard geometry characterized by an S—S torsion angle of −70°. This adjustment to accommodate the disulfide bridge causes the SdeA DUB backbone from residues 114 to 122 to adopt a different position with respect to the other two structures, which overlap nearly perfectly with each other (Supplementary Fig. S2*b*). The cost of this rearrangement is the unfurling of one hydrogen-bonding interaction in the SdeA DUB active-site area, at the N-terminal end of the helix carrying the catalytic cysteine (Fig. 2 ▸ *d* and Supplementary Fig. S2*c*). Thus, the disulfide complex shows that the interactions of SdeA DUB with Ub largely remain the same, except for a local distortion involving the active-site cysteine.

### 3.4. Crystal structure of OtDUB disulfide-linked to the ubiquitin G76C mutant

**Figure 4:**
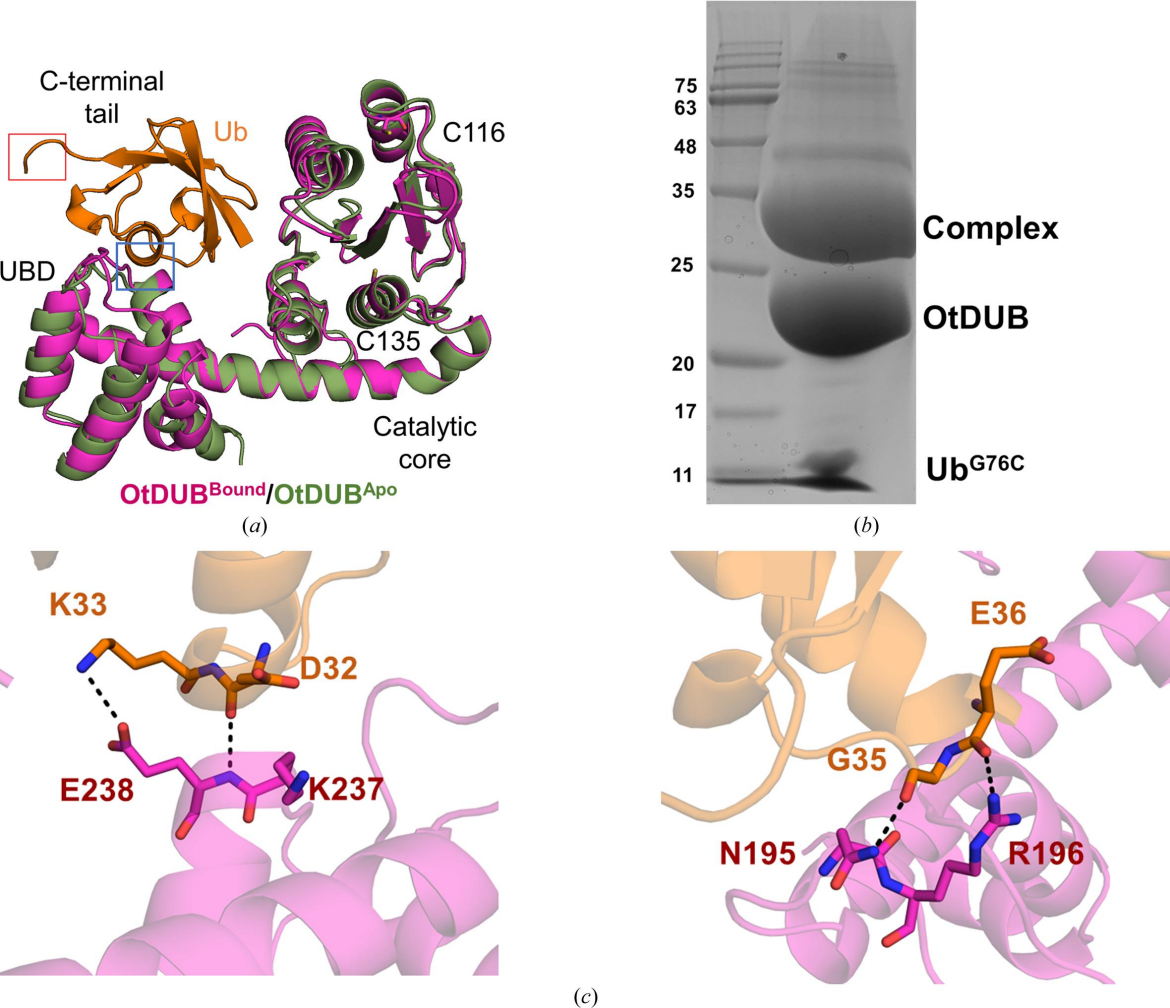
Structure of the disulfide-linked OtDUB–Ub complex. (*a*) Ribbon representation of the asymmetric unit of the crystal structure of Ub^G76C^ (orange) in complex with OtDUB (magenta) superimposed on the apo OtDUB structure (PDB entry 6upw, olive). The C-terminal tail of Ub is indicated by a red box. The blue box highlights the Ub-binding patch depicted in (*c*). (*b*) SDS–PAGE (nonreducing) analysis of the protein solution utilized for crystallization. (*c*) Residues from the UBD in OtDUB that interact with Ub residues enclosed in the blue box in (*a*) are shown in stick representation. Hydrogen bonds are indicated as dashed lines.

To further study the effectiveness of disulfide chemistry to characterize DUB binding interfaces, we decided to use the bacterial effector OtDUB, which has recently been crystallized with Ub in a noncovalent complex (Berk *et al.*, 2020 ▸). The Ub^G76C^–TNB adduct described previously was mixed with OtDUB in near-equimolar proportions and left overnight at room temperature, which yielded approximately 79% conversion, and the mixture was subjected to crystallization without further purification (Supplementary Fig. S3 and Fig. 4 ▸ *b*). A sample of the reaction mixture used for crystallization showed a single Ub adduct on nonreducing SDS–PAGE, while a portion of the OtDUB protein sample remained unmodified along with some Ub^G76C^ derivative. The OtDUB construct, unlike SdeA DUB (which only contains the catalytic cysteine), contains two additional cysteines (Cys111 and Cys116) beside the catalytic cysteine (Cys135), all of which are present in the core catalytic domain of the protein. By adding the Ub reagent in a limited stochiometric amount, we expected to capture a disulfide adduct involving primarily the catalytic cysteine.

**Figure 5:**
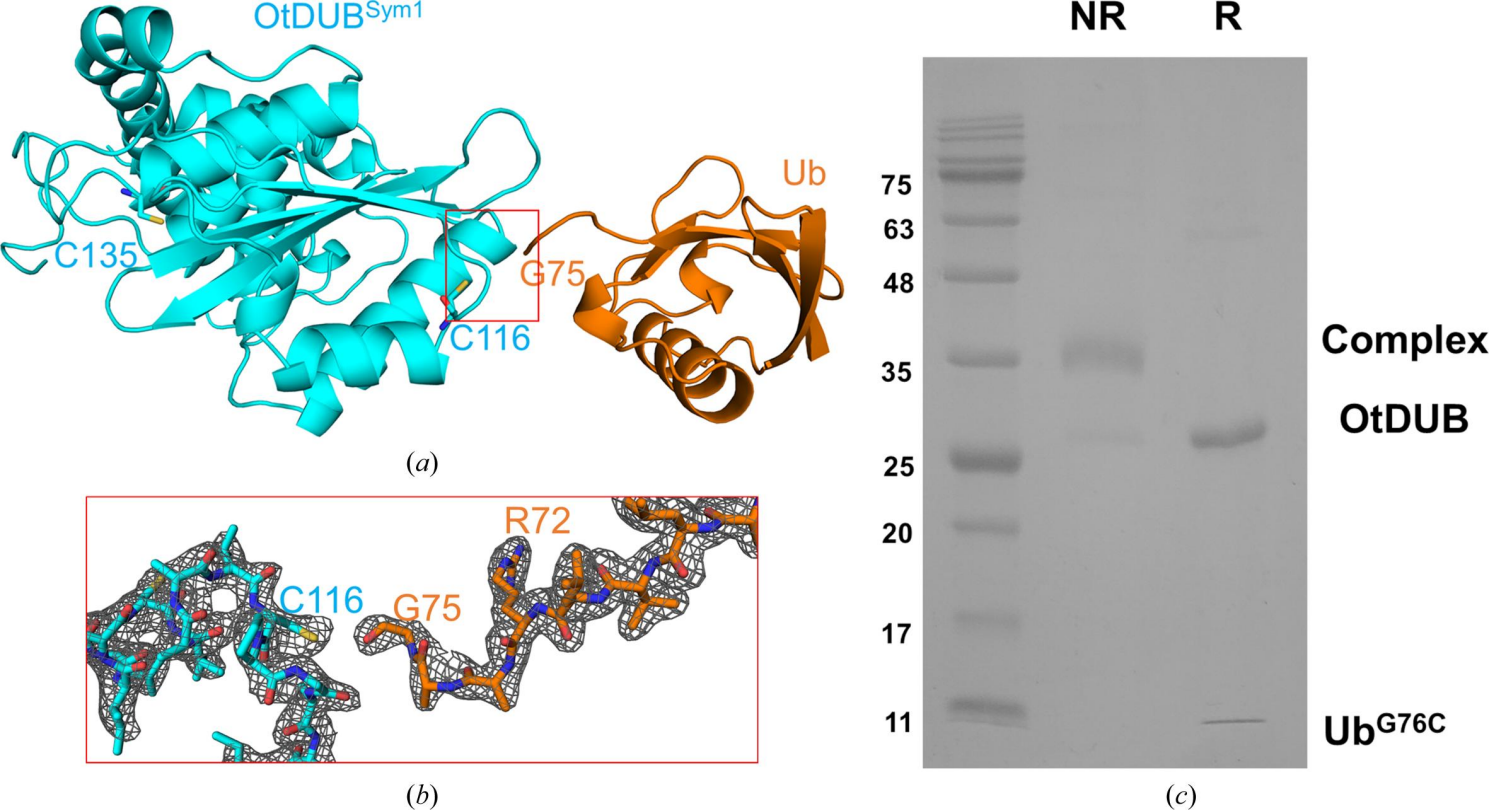
A likely disulfide bond connects OtDUB to ubiquitin from a symmetry-related counterpart. (*a*) A symmetry-related molecule of OtDUB (OtDUB^Sym1^, cyan) located proximal to the C-terminal tail of Ub from the asymmetric unit. Cys116 is shown in stick representation (orange). (*b*) An enlarged view of the boxed region showing the residues and the corresponding electron density (2*F*_o_ − *F*_c_ at 1σ) of the C-terminal end of Ub^G76C^ and a polypeptide segment around Cys116 of OtDUB. (*c*) Samples of the crystals were run on an SDS–PAGE gel after dissolving them in both nonreducing (NR) and reducing (R) SDS loading buffers.

Even though the protein sample remained as a mixture of at least three different species (Supplementary Fig. S3), crystallization trials successfully produced crystals that diffracted to 1.85 Å resolution. The crystals belonged to the orthorhombic space group *P*22_1_2_1_ (Table 1 ▸), with one complex comprising an OtDUB molecule and an Ub molecule in the asymmetric unit. The structure was solved by molecular replacement using OtDUB and Ub as two separate search models (see Section 2). The MR solution revealed a model of the asymmetric unit that contained a Ub next to an OtDUB, but with its C-terminus pointing away from the catalytic cysteine residue and with no disulfide linkage to any other cysteine in the same OtDUB chain (Fig. 4 ▸ *a*; PDB entry 8efx). Instead, the Ub C-terminal tail points towards a symmetry-related OtDUB neighbor, approaching its Cys116 (Fig. 5 ▸ *a*). (In OtDUB, this cysteine is nearly 10 Å from the catalytic cysteine.) However, the C-terminal tail of Ub^G76C^ was disordered after Arg72, with missing density for the side chains of Leu73 and Arg74, even though the backbone is traceable up to Gly75 (Fig. 5 ▸ *b*). While the terminal cysteine residue could not be placed due to missing density, the proximity and orientation of the C-terminus indicated that Ub^G76C^ could be disulfide-linked to Cys116 of the symmetry-related neighbor. Alternatively, despite the substantial population of a disulfide complex in solution, we might have managed to crystallize a non­covalent complex formed from the unreacted OtDUB and Ub^G76C^ species remaining in the reaction mixture. In probing this further, we harvested crystals from the same batch as used for diffraction, carefully washed them and subjected them to SDS–PAGE under either reducing or nonreducing conditions. Under the latter conditions, a single species corresponding to a covalent complex was detected, while the same dissolved crystals produced two distinct bands under reducing conditions (in the presence of DTT) corresponding to separated OtDUB and Ub chains, as expected for a disulfide-linked adduct (Fig. 5 ▸ *c*). Within the complex representing the asymmetric unit, OtDUB makes only a handful of contacts with Ub, leaving most of its canonical interaction patches (the widely used Ile44 and Ile36 patches as well as the C-terminus) unsatisfied. The few contacts with Ub in the asymmetric unit complex are provided entirely by four residues in the ubiquitin-binding domain (UBD; see below), but from a face diametrically opposite to a patch of residues known to be responsible for the high-affinity interaction with Ub (Berk *et al.*, 2020 ▸; Fig. 4 ▸ *a*). The intra-asymmetric unit contacts between the UBD and Ub utilize Lys33 and the backbone atoms of the Gly35-Glu36 segment of Ub and Asn195, Arg196, Lys237 and Glu238 of OtDUB (Fig. 4 ▸ *c*).

#### 3.4.1. Crystallographic contacts mediated by high-affinity interaction between Ub and OtDUB UBD

**Figure 6:**
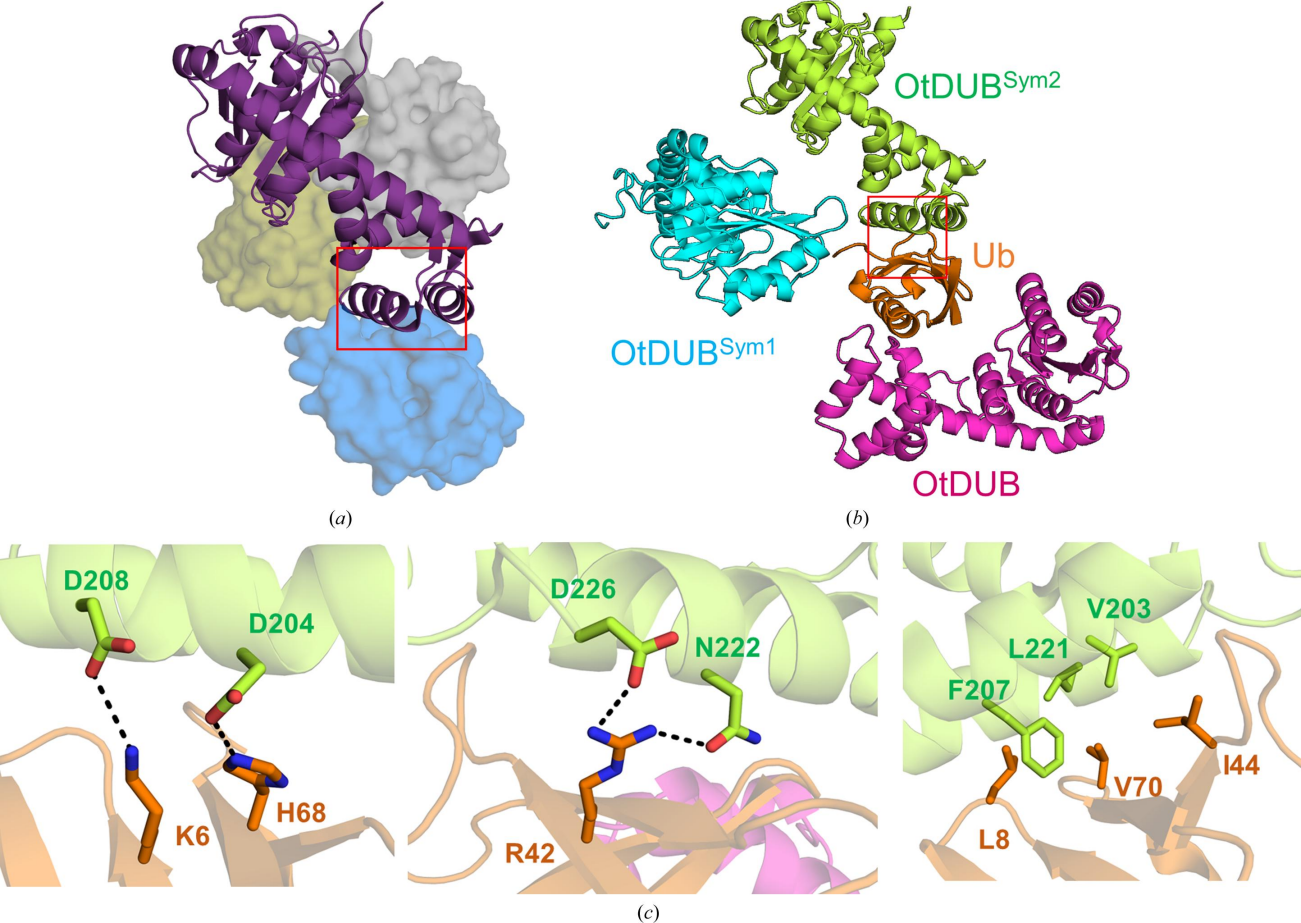
Interactions of UBD and Ub as packing contacts in the crystals. (*a*) The previously solved OtDUB structure in complex with three Ub molecules shown for comparison (PDB entry 6upu). OtDUB is depicted in ribbon representation and Ub is shown as a surface rendition. (*b*) The OtDUB–Ub^G76C^ complex (magenta and orange) in the asymmetric unit with two of its symmetry-related OtDUB molecules flanking Ub: OtDUB^Sym1^ (cyan) and OtDUB^Sym2^ (lime green). (*c*) Residues from the UBD domain of OtDUB^Sym2^ interact with the Ile44 patch of Ub^G76C^ of the asymmetric unit complex.

The likely inter-subunit disulfide in the crystals prompted us to further examine the crystallographic contacts (Fig. 6 ▸ *a*). In the previously characterized noncovalent complex that was produced under conditions of excess Ub, OtDUB showed three distinct Ub-binding sites: two of them belong to the catalytic domain, with Ub occupying the S1 and S2 sites (Fig. 6 ▸ *b*; the S1, S2 notation follows a similar nomenclature as that used to describe protease–substrate interactions, except that the S1, S2 *etc.* sites refer to the binding pocket for an entire Ub in the context of a polyubiquitin substrate; the first Ub preceding the scissile peptide bond binds at the S1 site, the Ub before that at the S2 site and so on), whereas the third Ub-binding site is contributed solely by a C-terminal extension after the catalytic domain. The extension, consisting of four α-helices, folds into a distinct domain that has been shown to function as an accessory module for recruiting Ub (accordingly, it is referred to as the ubiquitin-binding domain; UBD) through inter­actions amounting to single-digit nanomolar affinity (Berk *et al.*, 2020 ▸). The ubiquitin-binding site on the UBD does not fit the description of a substrate-like arrangement of Ub binding as represented by the S*i* or S*i*′ notation (*i* = 1, 2, 3 and so on). Moreover, this domain seems to fold independently of the catalytic domain and retains high-affinity Ub binding even as a separate construct outside the context of the OtDUB protein (Berk *et al.*, 2020 ▸).

The high-affinity UBD patch, while unsatisfied within the asymmetric unit complex, interacts with Ub from a different symmetry mate using all of the expected Ub–UBD inter­actions that constitute the high-affinity binding (Fig. 6 ▸ *c*). For example, the Ile44 patch on Ub^G76C^ (residues Leu8, Ile44 and Val70) makes close contact with a complementary hydrophobic patch of OtDUB UBD (Val203, Phe207 and Leu221), while electrostatic attraction brings Lys6, Arg42 and His68 of UbG76C into close contact with three aspartates (Asp204, Asp208 and Asp226) of UBD located within the high-affinity patch. The Ile44 interaction with the UBD hydrophobic patch brings interacting hydrophobic residues into close van der Waals contact (Supplementary Fig. S4*b*). Thus, the Ub–UBD high-affinity interaction provides crystal-packing contacts that seem to be important for crystal growth along one of the axes of the unit cell.

## 4. Discussion

Disulfide cross-linking has been used in the past to obtain structures comprising two or more proteins. In the ubiquitin field, this has been used to capture at least six Ub-bound structures of ubiquitin-conjugating enzymes and E3 ligases (Lorenz *et al.*, 2016 ▸; Wiener *et al.*, 2012 ▸; Maspero *et al.*, 2013 ▸; Kamadurai *et al.*, 2013 ▸; Jäckl *et al.*, 2018 ▸; Puvar *et al.*, 2020 ▸). In these examples, the disulfide link involves the catalytic cysteine of the ubiquitin-conjugating E2 enzyme or a HECT-family ubiquitin E3 ligase and the cysteine residue of the G76C Ub mutant. Here, we explore whether a similar strategy can be used to capture Ub bound to cysteine-protease DUBs. Using a panel of eukaryotic and prokaryotic DUBs, we find that the disulfide reactivity with Ub^G76C^ can show significant variation. In some cases, the reaction can proceed to a substantial degree, allowing the isolation of a single-Ub adduct of the disulfide-linked DUB for structural characterization, as exemplified by the CE-clan SdeA DUB from *L. pneumophila*, which contains a single cysteine as its catalytic residue. LotB, an OTU family DUB from the same organism, reacts readily but produces two distinct apparently single-Ub adducts. These species could result from the reaction of one Ub^G76C^ group with either the catalytic or another reactive cysteine. Previous studies on LotB (also known as Ceg23) have shown that in addition to the catalytic cysteine there is still at least one other reactive cysteine (Ma *et al.*, 2020 ▸), which could explain our observation of two distinct single-Ub adducts (Fig. 1 ▸ *c*). To the extent that the disulfide reaction is a proximity-driven effect, the observation of multiple disulfide modifications may indicate the presence of additional Ub-binding sites other than the expected S1 site.

While the previous structures of Ub bound to SdeA DUB (Sheedlo *et al.*, 2015 ▸, 2021 ▸) had many similarities to the disulfide-linked structure obtained in this study, this new structure shows a different arrangement of the catalytic cysteine and some nearby residues in the DUB active site (Fig. 2 ▸ *d* and Supplementary Figs. S2*b* and S2*c*). Compared with the Ub-bound noncovalent complex (representing binding of the Ub product) and the Ub-VME-bound structure, in which the electrophile warhead mimics the last glycine and the scissile peptide bond in a more or less isosteric arrangement, reaction with Ub^G76C^ results in local distortion at the active-site cysteine, presumably to accommodate the non-isosteric cysteine substitution at Gly76 (Supplementary Fig. S2*b*). In fact, this substitution may prevent the Ub mutant binding to the DUB in the first place, which could explain the lack of reaction with some of the other DUBs in our panel. It seems likely that SdeA DUB and those that readily react with the Ub mutant may possess an active site that is flexible enough to accommodate the cysteine substitution. The active-site cysteine in the disulfide-linked structure adopts an unproductive orientation which lies beyond an interacting distance from the catalytic histidine (S^γ^–N^δ^ distance of 7.7 Å). Thus, it appears that disulfide formation occurs when the cysteine is in a more accessible conformation while pointing away from the catalytic histidine, even though it is at the expense of the interaction that is observed in productive cysteine proteases (typically characterized by an S^γ^–N^δ^ distance of ∼4 Å). As an implication for structural analysis, the disulfide-linked structure on its own may not yield a productive arrangement of the catalytic residues of the DUB.

In contrast to SdeA DUB, the disulfide reaction of OtDUB with Ub76C resulted in an adduct that involves a different cysteine to the catalytic residue (Fig. 5 ▸). It is possible that some amount of catalytic cysteine-linked disulfide adduct could be produced in the reaction but did not yield crystals. Since we went straight from the reaction to crystallization without purification, our results may not entirely reflect the situation in solution. Nevertheless, the crystals were disulfide-linked, based on the behavior of the dissolved crystals under reducing conditions (Fig. 4 ▸ *c*), and the disulfide did not involve the catalytic cysteine but instead involved a surface-exposed cysteine.

Under conditions of a limiting concentration of the Ub reagent, it is possible that the nanomolar UBD-binding site becomes saturated first and it is this Ub-bound species that undergoes disulfide chemistry. The C-terminal tail of this Ub connected to the UBD in one complex may favor an ‘intermolecular’ reaction with a readily accessible cysteine of a different OtDUB–Ub complex in solution (over an ‘intra­molecular’ reaction with the catalytic cysteine, for example, within the same complex), giving rise to the crystals that we have characterized here. In retrospect, it might have been possible to capture the S1 binding site through disulfide linkage only if we had used an excess of the Ub reagent.

The two examples of disulfide strategy described here illustrate the potential limitations of this approach compared with co-crystallization with Ub C-terminal electrophiles. In spite of requiring more steps in their production, in contrast to the simpler cysteine mutagenesis involved in the disulfide strategy, the Ub electrophiles are more likely to produce biologically relevant complexes.

## Supplementary Material

PDB reference: SdeA DUB disulfide cross-linked with ubiquitin, 8efw

PDB reference: OtDUB disulfide cross-linked with ubiquitin, 8efx

Supplementary Figures. DOI: 10.1107/S2059798323008501/nj5320sup1.pdf

## Figures and Tables

**Table 1:** Data-collection and processing statistics Values in parentheses are for the outer shell.

	SdeA DUB^1–200^–Ub^G76C^	OtDUB^1–259^–Ub^G76C^
Wavelength	0.97918	0.97918
Temperature (K)	100	100
Resolution range	39.36–2.81 (2.91–2.81)	49.68–1.85 (1.92–1.85)
Space group	*P*3_2_21	*P*22_1_2_1_
*a*, *b*, *c* (Å)	90.905, 90.905, 65.821	42.399, 83.008, 99.354
α, β, γ (°)	90, 90, 120	90, 90, 90
Total reflections	419149 (7869)	196183 (1035)
Unique reflections	7838 (763)	30705 (1788)
Multiplicity	53.0 (2.8)	6.4 (5.8)
Completeness (%)	98.80 (99.61)	99.40 (95.40)
Mean *I*/σ(*I*)	21.4 (2.5)	11.2 (0.9)
Wilson *B* factor (Å^2^)	69.85	36.54
*R*_meas_	0.244 (1.195)	0.097 (1.798)
*R*_p.i.m._	0.138 (0.494)	0.051 (0.981)
CC_1/2_	0.980 (0.363)	0.998 (0.409)
Reflections used in refinement	7829 (762)	30626 (2998)
Reflections used for *R*_free_	781 (79)	1995 (195)
*R*_work_	0.2421 (0.3777)	0.1976 (0.3284)
*R*_free_	0.2860 (0.4011)	0.2291 (0.3440)
No. of non-H atoms		
Total	1851	2705
Macromolecules	1851	2544
Solvent	0	161
Protein residues	259	334
R.m.s.d., bond lengths (°)	0.008	0.007
R.m.s.d., angles (°)	1.42	0.79
Ramachandran favored (%)	90.00	97.27
Ramachandran allowed (%)	7.00	2.73
Ramachandran outliers (%)	3.00	0.00
Rotamer outliers (%)	0.00	0.00
Clashscore	21.68	4.34
Average *B* factor (Å^2^)		
Overall	64.21	32.98
Macromolecules	86.37	43.07
Solvent	0	48.23
