# Supplementary material for: Cocrystallization of ubiquitin–deubiquitinase complexes through disulfide linkage
Source: Acta Crystallogr D Struct Biol. 2023 Oct 25;79(Pt 11):1044–55. doi: 10.1107/S2059798323008501 (PMC10619426; doi:10.1107/S2059798323008501)
Supplement: Supplementary file 1 [file d-79-01044-sup1.pdf]

STRUCTURAL BIOLOGY

Volume 79 (2023)

Supporting information for article:

Cocrystallization of ubiquitin–deubiquitinase complexes through disulfide linkage

Kristos I. Negron Teron and Chittaranjan Das

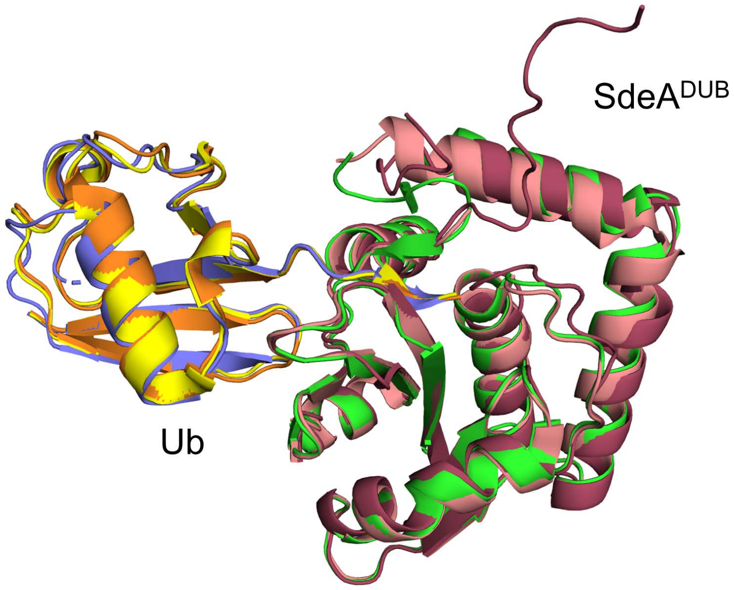

Figure S1 Comparison of all three SdeA DUB-Ub complex structures. Ribbon representation of all three Ub bound SdeA DUB structures: SdeA DUB bound to Ub^{G76C}, SdeA DUB bound to Ub-VME, and SdeA DUB bound to the Ub product (PDB: 8EFW, 5CRA, and 6WTG respectively).

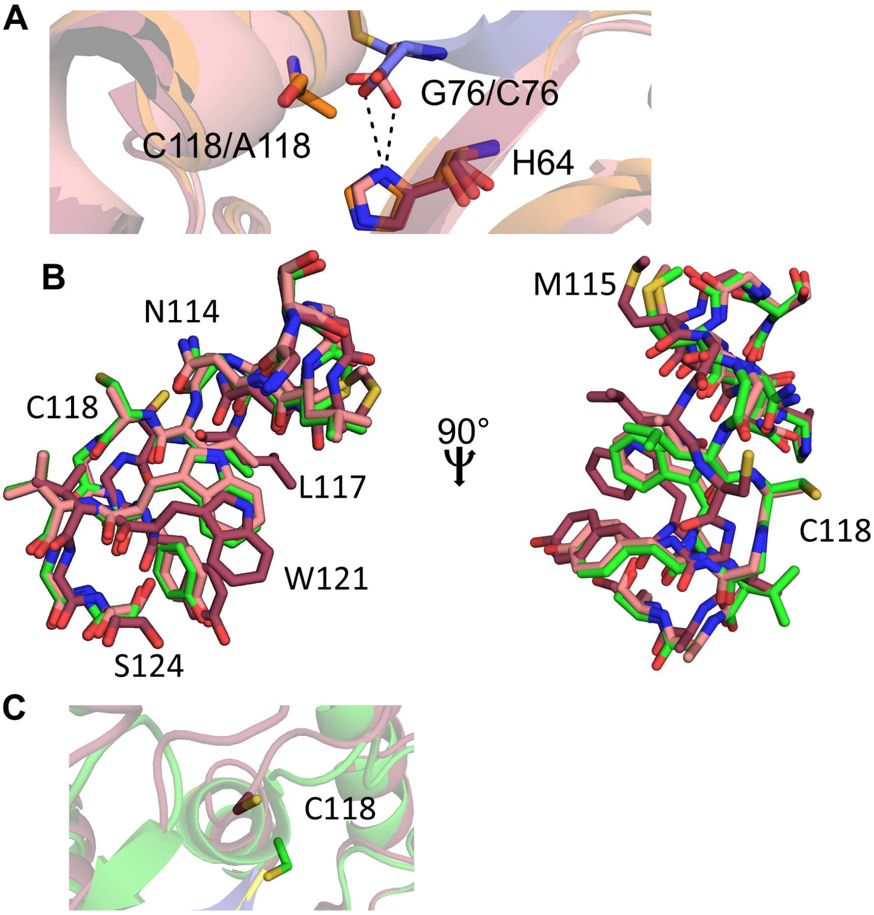

Figure S2 Local changes and similarities between the different SdeA DUB structures**. A**, The terminal carboxylate group of Ub^{G76C} in the disulfide-linked complex maintains a similar orientation and hydrogen-bonding interaction with the catalytic histidine of SdeA DUB to the Ub product bound complex. **B**, Shift in local residues near the catalytic cysteine of SdeA DUB observed in the disulfide-linked complex compared to the VME bound and product bound structures. **C**, Superposition of Ub-VME bound and the disulfide-linked SdeA DUB Ub^{G76C} complex structure shows unfurling of one helical turn that carries the catalytic cysteine to accommodate the disulfide bridge between C76 of Ub^{G76C} and Cys118 of SdeA DUB. The cysteine in the SdeA Ub-VME complex represents its native-like conformation.

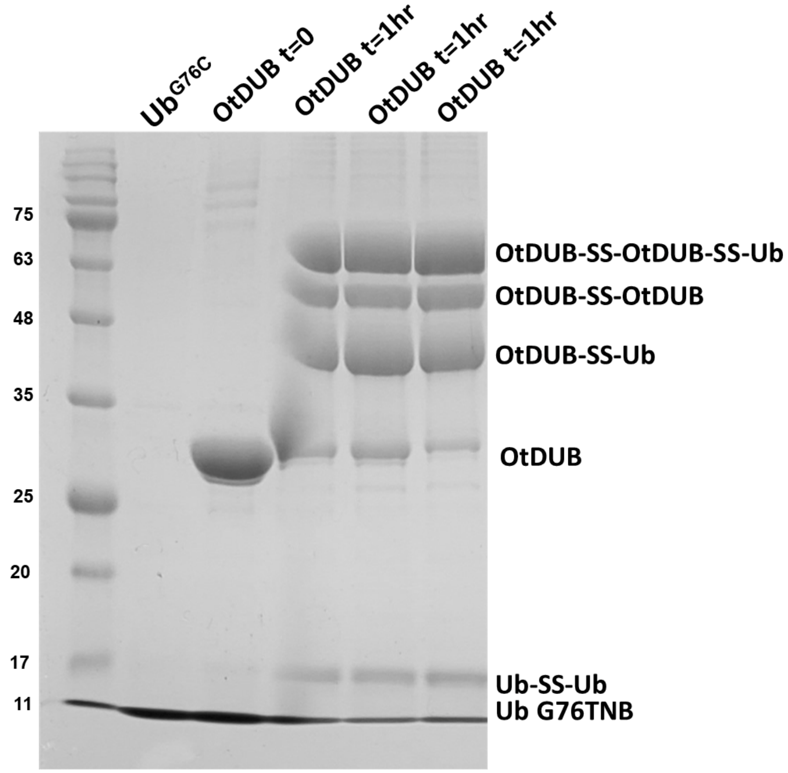

Figure S3 Conversion of OtDUB to DUB-Ub complex. SDS-PAGE (non-reducing) analysis of the reaction mixture of OtDUB and Ub^{G76C}. The conversion of complex was further analyzed using ImageJ software.

A

SdeA residue	Ub residue	Distance
E9	R74	2.7Å
S29	L8	3.3Å
Y33	Q40	3.0Å
P58	Q40	2.9Å
D61	Q40	2.7Å

B

OtDUB residue	Ub resudue	Distance
N195	G35	2.7Å
R196	E36	3.1Å
V203	I44	4.5Å
D204	H68	2.8Å
F207	L8	4.1Å
D208	K6	3.3Å
L221	V70	4.1Å
N222	R42	2.8Å
D226	R42	3.3Å
K237	D32	2.9Å
E238	K33	3.3Å

Figure S4 Distance measurements between Ub and the binding interfaces found on both the SdeA and OtDUB disulfide bridged structures**.** **A**, Table representing the distances between the SdeA DUB domain and Ub measured in angstroms. **B**, Table representing the distances between the UBD domain of OtDUB and Ub measured in angstroms.
